# Myxoid Liposarcomas of the Thigh: Pre-Operative Presentation, Clinical Outcomes, and Functional Results of Surgical Treatment

**DOI:** 10.3390/healthcare12171718

**Published:** 2024-08-28

**Authors:** Edoardo Ipponi, Elena Bechini, Martina Cordoni, Fabrizia Gentili, Fabio Cosseddu, Antonio D’Arienzo, Lorenzo Andreani

**Affiliations:** Department of Orthopedics and Trauma Surgery, University of Pisa, 56126 Pisa, Italy

**Keywords:** soft tissue sarcoma, radiotherapy, resection margins, tumor grade, MSTS, complications

## Abstract

Myxoid liposarcomas are malignant soft-tissue sarcomas whose treatment represents a challenge, even for the most experienced surgeon. In this study, we report on our experience with the treatment of myxoid liposarcomas of the thigh. Our retrospective analysis included myxoid liposarcomas of the thigh treated with surgical resection between 2016 and 2022. Resection margins, complications, local recurrences, and metastases were recorded. The oncological outcome of each case was evaluated at their latest follow-up. Adjuvant therapies were administered according to the ESMO guidelines. Functionality was assessed with the MSTS score before surgery and at the patients’ latest follow-up. Thirty cases (ten high-grade and 20 low-grade) were included. The mean diameter was 11.8 cm. Twenty-four cases had wide margins (80%) and six (20%) were marginal. Five cases (60% marginal) had local recurrences (17%). Marginal resection was associated with a higher risk of local recurrence (*p* = 0.041). Three cases with high-grade tumors (10%) developed metastases. At the patients’ latest follow-up, their mean MSTS score had risen from 22.9 to 27.3. While tumor grade influences the risk of metastases, the quality of resection margins can determine the local recurrence rate. An adequate surgery can lead to good post-operative functional outcomes.

## 1. Introduction

Liposarcoma is one of the most common sarcomas found in adults, and it can be defined as a mesenchymal malignancy characterized by adipocyte differentiation [1,2]. Myxoid liposarcomas (MLs) are one of the subtypes of liposarcomas recognized by the 2020 WHO classification for soft-tissue sarcomas [2]. This classification, based on pathological findings including immunohistochemical and genetic features, divides liposarcomas into well-differentiated, dedifferentiated, myxoid, and pleomorphic liposarcomas. Histologically, all subtypes of liposarcomas share the presence of at least some cells that bear a resemblance to the normal fat cells of the common adipose tissue. MLs in particular are characterized by myxoid areas, with a rich capillary-size vascular network set in the myxoid stroma and small, round, or oval neoplastic cells with minimal cytoplasm [2,3]. Accounting for approximately 10–20% of all liposarcomas, MLs predominantly affect adults between 30 and 60 [3]. The mediastinum represents the most common anatomic site, followed by the limbs and the head and neck region [2].

Clinically, liposarcomas of the thigh frequently present as painless, slowly enlarging masses. Due to their indolent nature, these tumors can remain undetected for months or even years. Despite their often slow growth, MLs can be prone to local aggression towards the nearby healthy tissues and metastasize to distant body segments [1,2,3].

Once the histological diagnosis has been established, the primary treatment for myxoid liposarcoma is represented by surgical resection aimed at achieving wide margins to minimize the risk of local recurrence. Neoadjuvant radiation therapy can be considered for wide lesions, especially if they are larger than 10 cm, and those who already have a proven metastatic disease [4]. Adjuvant radiotherapy can also be an effective adjuvant treatment, especially in cases where wide surgical margins cannot be obtained. Chemotherapy may be considered for advanced or metastatic disease, although its efficacy is variable, and the optimal regimen remains controversial [4,5,6,7,8]. 

Our study aimed to evaluate the clinical and functional outcomes of patients with myxoid liposarcomas of the thigh treated with surgical resection. We also assessed the impact of pre-operative and intra-operative conditions as potential prognostic factors in a mid-to-long-term scenario.

## 2. Materials and Methods

This single-center retrospective study was performed in accordance with the ethical standards set in the 1964 Declaration of Helsinki and its later amendments. All patients gave their written consent. Our study consisted of a review of all the patients who were diagnosed with a primary myxoid liposarcoma of the thigh and were treated with surgical resection in our institution between January 2016 and December 2022.

We collected personal data for each case, including age at diagnosis and gender. Each case underwent a careful evaluation of their total body CT scans and thigh MRI images. CT scans were used to assess the systemic spread of the disease, and only cases without metastases at the moment of their diagnosis (Stage I or II according to the Enneking classification system) were included in our study. MRI images were used to localize the lesion, orientate diagnosis, guide surgical planning, and estimate the tumor size (larger diameter calculated in centimeters). Only cases with certain histological diagnoses of myxoid liposarcoma of the thigh were included in our study. The histological diagnosis was established with an ultrasound-guided needle biopsy, and later confirmed on surgical specimens. For each case, we recorded the diagnostic interval, defined as the time intercurred between the detection of the first symptoms and the moment in which diagnosis was established. The pre-operative functional status of all patients was assessed using the Musculoskeletal Society (MSTS) scoring system.

We recorded the date and the type of surgeries performed. Excision was performed with wide macroscopical margins. The surgical approach was planned considering the localization, the extension, and the anatomical relationships between the neoplasm and the nearby vascular and nervous noble structures. The latter were routinely identified and protected intraoperatively to prevent iatrogenic damages. All the surgical specimens were sent for histological examination by a pathologist to confirm the diagnosis of myxoid liposarcoma, calculate its size, and assess the grade of malignancy. One or more surgical drainages were set in place while closing the surgical bed and removed once the drainage had stopped or had become less than 25 mL/day. At the end of the intervention, wounds received closed incisional negative-pressure wound therapy (ciNPWT) or compressive wound dressings to prevent the onset of post-operative complications, such as seromas or hematomas.

Our patients received chemotherapy and radiation therapy according to the latest ESMO guidelines for the multidisciplinary approach to myxoid liposarcomas [6]. Pre-operative radiotherapy (RT) was performed for those cases who had a diagnosis of high-grade liposarcoma after the needle biopsy. Post-operative RT was performed in patients with high-grade disease on their definitive histological evaluation or in case of marginal or intralesional resections. The post-operative follow-up consisted of several outpatient office visits, local MRIs, and global imaging evaluations alternating chest–abdomen CT scans with chest X-rays and abdomen ultrasounds. The first clinical evaluation was performed 30 days after surgery in order to evaluate the state of the surgical wounds and patients’ global clinical conditions.

Further clinical evaluations, associated with MRIs of the thigh and imaging of chest and abdomen, were performed within three months after surgery, and repeated every three months for two years, then twice per year for the following three years, and finally on a yearly basis. Local recurrences and distant metastases, confirmed by imaging findings and histological analysis, were recorded. The oncologic status of each patient was assessed at their latest follow-up as follows: continuously disease-free (CDF), no evidence of disease (NED), alive with disease (AWD), died of disease (DOD), or died of other causes (DOC).

Each complication with grade II or higher according to the Clavien–Dindo Classification was recorded, as well as their treatments.

The patients’ post-operative functional status was assessed using the Musculoskeletal Society (MSTS) scoring system.

Statistical analysis was performed using Stata SE 13 (StataCorp LLC, College Station, TX, USA). Statistical significance was set at 0.05 for all endpoints.

## 3. Results

Thirty-two cases of suffering from deep myxoid liposarcomas of the thigh were treated in our institution between January 2016 and December 2022. Two of them were lost to follow-up, while the other thirty patients remained in our study. They were 13 females and 17 males, with a mean age of 49.2 (17–77) years at surgery. All our cases had clinical signs attributable to the neoplastic mass, and none had an incidental diagnosis. All our cases noticed swelling in the area involved by the tumor, but only two (6.7%) of them complained of localized pain (VAS equal to or higher than 3) in the same region. On average, our patients came to our attention 7.6 (1–60) months after the onset of the first symptom. The lesions reduced patients’ pre-operative functional status by at least mildly, as testified by the mean Musculoskeletal Tumor Society (MSTS) score of 25.8 (15–29).

**Figure 1:**
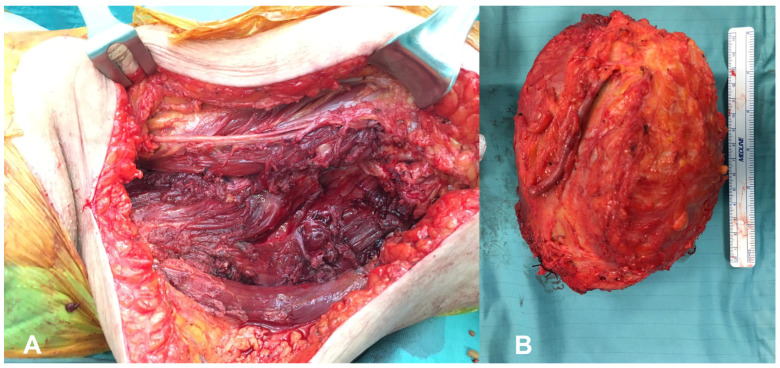
Intra-operative images of the surgical field after the resection of a voluminous myxoid liposarcoma of the medial-anterior thigh. (**A**) The femoral nerve, preserved, is displayed running horizontally in the upper area of the picture. The great saphenous vein had to be sacrificed and was removed alongside the surgical specimen, whose larger diameter exceeded 15 cm (**B**).

**Figure 2:**
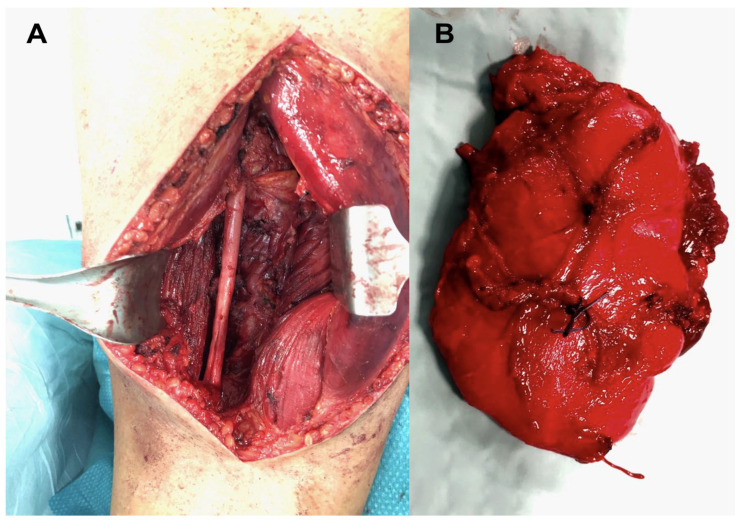
Intra-operative images of the resection of a myxoid liposarcoma of the posterior thigh, showing the continuity of the sciatic nerve (**A**) and the shape of the removed mass (**B**).

In 12 cases, the neoplasm involved the anterior compartment of the thigh. Ten patients had their neoplasms localized in the posterior compartment, and the remaining eight lesions occupied the medial compartment of the thigh. According to the pre-operative MRI images, the mean maximum diameter of the neoplastic masses was 11.9 (5–23) cm. In 18 of our 30 cases (60%), lesions were larger than 10 cm. None of our cases had metastases at the moment of the diagnosis. All cases received a complete excision of their neoplasm (Figure 1 and Figure 2).

Wide margins were obtained in 24 cases, whereas resections were marginal in the remaining 6 cases. In all the patients included in our study, histological evaluations on surgical specimens confirmed the diagnosis of myxoid liposarcoma. 

Ten cases were diagnosed with high-grade myxoid liposarcomas, whereas the remaining twenty cases had low-grade lesions.

None of our cases had significant intraoperative complications. In the first days after surgery, 16 of our 30 cases had compressive wound dressings, while the remaining 14 cases received closed incisional negative-pressure wound therapy (ciNPWT).

**Figure 3:**
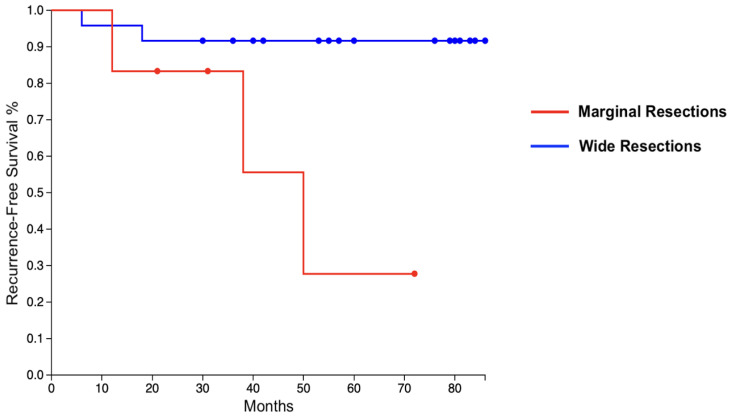
A Kaplan–Meyer graph that shows the recurrence-free survival of our population, divided into cases who had marginal resections (red) or wide resections (blue).

The mean postoperative follow-up of our population was 58.1 (20–89) months. At their latest follow-up, 21 of our 30 cases (70%) were continuously disease-free (CDF). One case (3%) died of other causes 49 months after surgery, but did not suffer from local recurrences or metastasis. Five cases (17%) had local recurrences, which were diagnosed on average 24.8 (6–50) months after surgery. Only one of these five cases had a high-grade sarcoma, while the remaining four suffered from low-grade lesions. Therefore, Fisher’s Exact Test affirmed that, in our population, low-grade sarcomas had a significantly higher correlation with local recurrences in our cohort (*p* = 0.031). From a surgical point of view, resection had been carried out with wide margins in only two of five cases. According to Fisher’s Exact Test, local recurrences were significantly more common in those who had received marginal resections (*p* = 0.041). A figurative representation of local recurrence-free survival is provided in Figure 3.

Four of those patients who had local recurrences underwent further surgical resection and no evidence of disease (NED) at their latest follow-up. The remaining patient is alive with disease (AWD) as his surgical intervention still had to be performed at the moment of our data collection. Three cases (10%) did not develop local recurrences, but have been diagnosed with metastases in distant sites. All of them had been diagnosed with high-grade myxoid liposarcomas, which were therefore statistically associated with more metastases compared with low-grade lesions (Fisher’s Exact Test, *p* = 0.030). One of those who had metastatic lesions was successfully treated with a pulmonary metastasectomy and showed no evidence of disease (NED) at his latest follow-up. Two other cases had metastases in multiple localizations; one of them was alive with disease at the moment of our data collection, whereas the other died of disease.

Although our data did not highlight a significant correlation between tumor size and local recurrence or metastasis, a Pearson correlation test reported that patients who were continuously disease-free at their latest follow-up had a significantly lower pre-operative lesion size than other patients (*p* = 0.047).

Postoperative complications occurred in six patients (20%). Two patients (7%) developed seromas. Both cases were successfully treated with ultrasound-guided percutaneous drainage. Two other cases (7%) suffered from superficial infections, which were treated with surgical debridement and antibiotic therapy. Wound debridement was also performed for one case (3%) that suffered from wound dehiscence. One patient, treated for a myxoid liposarcoma of the anterior thigh, developed dysesthesia and hypoesthesia in the anterior thigh area in the absence of strength limitations. All these complications occurred within two months after surgery.

The mean MSTS score of our patients at their latest follow-up was 27.0 (15–30). This value was significantly higher than the one recorded pre-operatively, according to a two-tailed Student’s t-test (*p* = 0.030). Our cohort did not highlight significant correlations between post-operative functionality and size, location, surgical margins, or tumor grade.

A summary of our cohort, divided into low grades and high grades, is provided in Table 1.

## 4. Discussion

Liposarcoma is one of the most commonly diagnosed sarcomas in the adult population, comprising up to 12.8% of all soft-tissue malignancies. Previous studies showed higher incidences between the fourth and seventh decades of life, with a mean age at diagnosis between 49 and 50 years. The same authors reported a slightly higher incidence in males than females [3,9].

The clinical presentation of liposarcomas in the thigh is often nonspecific, and a long time can pass between the onset of the first symptoms and a severe suspicion of a neoplasm [10]. In a review published in 2020, Soomers et al. [11] reported that the mean diagnostic intervals for soft tissue sarcomas could vary between a minimum of 4 and a maximum of even 615 weeks. Our cohort of myxoid liposarcomas of the thigh came to a diagnosis on average 7.6 months after their first symptoms, data in line with the previously reported literature. Although all our cases complained of a swelling in their thigh, only two cases (6.7%) had localized pain. This confirms the assertions of the theory of Brouns et al. [12], who suggested that one of the main reasons for the diagnostic delay was having a painless mass, and the data provided by George and Grimer, who experienced a longer diagnostic delay for painful sarcomas [13]. However, the fact that all our patients had swelling in their affected thighs should raise the attention of general clinicians and orthopedic surgeons in territorial hospitals. This would reduce the risk of misdiagnosis and allow a progressive reduction in diagnostic intervals for the years to come.

Once the clinical picture and the first imaging evidence raise the suspicion of a soft tissue sarcoma, patients should be taken to referral centers to carry out the final steps of the diagnostic pathway. MRIs should be used to assess the size and the localization of the lesion, with a particular focus on the involvement of the nearby noble anatomical structures [14]. Surgeons should pay particular care to the sciatic and femoral nerve and the femoral vessels, which are essential for the post-operative functionality of the treated lower limb. The same images, matched with intra-operative ultrasound guidance, are also necessary to perform an adequate needle biopsy [15]. When performing a needle biopsy, physicians should focus on taking an adequate amount of tissue for the following histological examination, while choosing an entry point that will be later included in the subsequent surgical resection, in order to minimize the risk of tumor seeding [16,17]. The histological examination of the tissue obtained during biopsy, and later of the entire surgical specimen after surgical resection, represents the final point of the diagnostic pathway. Pathologists are called to establish the definitive diagnosis of MLs, ruling out the other possibilities in differential diagnosis [2,6,7,9,10]. Deacu et al. [18] proposed a histopathological scoring system useful both in differential diagnosis, as well as for the prediction of the immunohistochemical and the prediction of the immunohistochemical and genetic examinations.

Once the diagnosis has been established, a medical and surgical multidisciplinary evaluation of each case should be performed in order to decide on the best treatment for every single patient [6,7,19]. The therapeutic approach toward MLs in unspecialized facilities is a significant risk factor for local recurrence. Engström et al. [20] reported the outcomes of the Scandinavian sarcoma group for liposarcomas, presenting a 47% recurrence rate for liposarcomas operated in nonspecialized centers. Lemeur et al. [21] reported 23% local recurrence in a series with six myxoid liposarcomas treated initially in nonspecialized settings, four of which were managed with intralesional excision. High-volume referral centers reported lower risks of local recurrences. Killpatrick et al. [22] reported the experience of the Mayo Clinic, with a recurrence rate of 14% among their 91 cases (61 thighs) with myxoid and round cell liposarcomas. Fiore et al. [23] from the Italian National Cancer Institute in Milan and Muratori et al. [1] from the University of Florence had a local recurrence rate of 25% and 10%, respectively. The 17% rate of our cohort, consisting of 30 cases with MLSs localized exclusively in the thigh, was comparable to the ones obtained at the other oncological referral centers.

Excision plays a pivotal role in the treatment of neoplasms [1,6,7]. An en bloc resection with wide margins is theoretically essential to minimize the chances of leaving neoplastic cells in the surgical bed, thereby reducing the risk of local recurrence [24]. Adequate pre-operative planning and surgical expertise are necessary to prevent an intralesional approach and avoid the excessive sacrifice of muscular masses or the incidental lesion of the noble neurovascular structures in the thigh. Blunt dissection should be performed to respect the anatomical layers, limiting sharp approaches that could expose and create a breach in the tumor’s pseudocapsule [25,26]. When the neoplasm involves the femoral artery, surgeons are recommended to make a multidisciplinary approach with vascular surgeons to perform a vascular bypass and spare the vascularization of the lower limb while obtaining wide margins, rather than living tumor tissue in the surgical bed [25,26,27]. In our cohort, patients who had marginal resections had a significantly higher risk of developing local recurrences through their post-operative intercourse, confirming what had already been stated by Kilpatrick et al. [22] and Muratori et al. [1]. Our findings suggest that the quality of margins should be considered a pivotal factor for patients’ prognosis, and surgeons should always aim for wide reactions when approaching myxoid liposarcomas of the thigh, regardless of their histological grade. Unlike Lemeur et al. [21], our results did not show a significant correlation between the histological grade of the lesion and its recurrence risk. While histological grade did not represent a factor for local recurrence, our cases with high-grade lesions had a significantly higher risk of developing metastases. These data are consistent with what had already been reported by several studies in the modern literature, suggesting the higher tendency of high-grade lesions to spread systemically [1,3,4,9,10,11,12,14,15,16,17,18,19,20,21,22,23,28]. In our cohort, the impact of the histological grade was also hinted at by the fact that both our cases who died during their follow-up had a high-grade MLS. The association between histological grade and overall survival was not confirmed by statistical significance due to the limited size of our cohort. However, our overall survival of 93% was comparable to those in more extensive studies [1,3,4,9,10,11,12,14,15,16,17,18,19,20,21,22,23,28]. 

The relatively long life expectancy of patients treated with surgical resection on the thigh should put the focus on post-operative functional outcomes. Returning to the patient’s activities of daily living can be complex and full of pitfalls, especially for those with large lesions and who suffer from the sacrifice of a large share of the muscular masses or nervous structures [29,30,31,32]. At their latest follow-up, our patients had a mean MSTS score of 27 (10–30), with 27 out of 30 cases (90%) scoring 25 or higher. This value was significantly higher than those recorded pre-operatively, testifying to the effectiveness of the surgical approach in restoring the performances of treated thighs, despite the often extensive sacrifice of muscular masses. No correlations between the lesions’ size or localization and post-operative functionality emerged from our study, suggesting that also large-sized lesions could find significant benefit from an adequate surgical approach.

These results could be obtained with accurate pre-operative planning, a surgical approach that includes careful resection, preserving the healthy tissues and restoring the continuity of the anatomical layers in the treated compartments, and finally, adequate wound dressing [32,33,34]. Preserving healthy major nerves and vessels and sparing a sufficient share of muscular fibers in the anterior, medial, or posterior compartment allows for the maintenance of the neuromuscular units responsible for hip and knee movements [29,31,32,34]. In cases where the overall muscular continuity is preserved, muscles survive, and the post-operative muscular regeneration could afford patients a significant share of their pre-operative muscular strength [31,32,34,35]. Patients should, therefore, be encouraged and guided to a careful rehabilitation protocol that could support tissue healing and prevent the onset of joint stiffness. Following the modern concepts of rehabilitation in orthopedic surgery, surgeons and physiotherapists should aim for early mobilization of the hip and knee and progressive weight-bearing on the treated limb [35,36,37,38]. Surgeons should tailor the timing of the single steps, including mobilization without resistance in bed, walking with crutches and light weight-bearing, load progression, and later walking without aids as they depend on the clinical picture of every single case and the magnitude of the intervention they had [36,37,38,39]. Correct dialog between surgeons, patients, and physiotherapists is advisable to ease the process and maximize patient compliance.

We acknowledge the limitations of our study. The rarity of myxoid liposarcomas significantly limited the size of our population. This partially limited the statistical significance of some of the data associations we wanted to investigate at the beginning of our research. The retrospective nature of our research, which did not allow for the complete standardization of intra-operative and post-operative procedures for each patient, was another limitation.

Beyond these limitations, our study provides evidence about the effectiveness of the surgical approach for myxoid liposarcomas of the thigh, evaluating the significance of factors such as size, histological grade, and the quality of resection margins. In particular, a high tumor grade is associated with developing post-operative metastases, whereas the quality of resection margins has a more prominent correlation with local recurrence rates. Patients with pain and swelling of their thighs should undergo a correct diagnostic pathway, including pre-operative imaging evidence, eventually followed by careful biopsies and histological examinations to be performed in referral centers. Blunt dissection and respect for the anatomical barriers should guide surgeons to avoid direct contact with the neoplasm and maintain good quality resection margins, representing the surgical field’s main aim. In most cases, experienced and skilled surgeons could dovetail the complete resection of the neoplasm with the sparing of a relatively large share of muscular tissue, which could heal and allow functional recovery after adequate rehabilitation. The good overall free and global survival after surgery attests to the effectiveness of surgical and multidisciplinary approaches, which are not limited to surgical and medical treatments but also rely on daily mobilization and physical therapy. An adequate surgical resection and careful management of the remaining healthy soft tissues can lead to good functional outcomes, allowing for most patients to return to their activities of daily living and restoring a higher quality of life.

## 5. Conclusions

In conclusion, our outcomes suggest that surgery, associated with proper neoadjuvant and adjuvant therapies when necessary, can be effective in treating myxoid lipomas of the thigh with the aim of eradicating the tumor and restoring patients’ lower limb performances. Good life expectancy and satisfactory post-operative performance can lead treated patients to long and relatively high-quality lives in the months and years to come.

## Figures and Tables

**Table 1:** This is a schematic summarization of our cohort. It shows data of our cohort evaluated as a whole and sorted per histological grading. Statistically significant differences between high-grade and low-grade populations are reported in column *p* on the right.

	Low-Grade (*n* = 20)	High-Grade (*n* = 10)	Total Population (*n* = 30)	*p* (<0.05)
**Number**	20	10	30	-
**Mean Age** (min–max)	45.5 (17–71)	56.7 (25–77)	49.2 (17–77)	-
**Gender** **Female** **Male**	11 9	2 8	13 17	-
**Average** **Diagnostic Interval** (min–max)	7.7 (1–16)	7.5 (1–60)	7.6 (1–60)	
**Localization** **Anterior Compartment** **Medial Compartment** **Posterior Compartment**	7 6 7	5 2 3	12 8 10	-
**Mean Size** (min–max)	11.2 cm (5–18)	13.2 cm (5–23)	11.9 cm (5–23)	-
**Pre-Op Swelling** (percentage)	20 (100%)	10 (100%)	30 (100%)	-
**Pre-Op Pain** (percentage)	2 (10%)	0	2 (7%)	-
**Mean Pre-Op** **MSTS Score** (min–max)	26.0 (15–29)	25.2 (15–29)	25.8 (15–29)	-
**Pre-Op** **Radiotherapy** (percentage)	0	8 (80%)	9 (27%)	-
**Margins** **Wide** **Marginal**	16 4	8 2	24 6	-
**Intra-Op** **Complications**	0	0	0	-
**Post-Op** **Complications** (percentage) **Seroma** **Infection** **Dehiscence** **Dysesthesia**	4 (20%) 1 1 1 1	2 (20%) 1 1 0 0	6 (20%) 2 2 1 1	-
**Post-Op** **Radiotherapy** (percentage)	3 (15%)	0	3 (10%)	-
**Local** **Recurrences** (percentage)	4 (20%)	1 (10%)	5 (15%)	FISHER’S EXACT TEST: Contingency between low grade and local recurrence (*p* = 0.041)
**Metastases** (percentage)	0	3 (33%)	3 (10%)	FISHER’S EXACT TEST: Contingency between high-grade lesions and metastases (*p* = 0.031)
**Oncological** **Outcomes** **CDF** (percentage) **DOC** (percentage) **NED** (percentage) **AWD** (percentage) **DOD** (percentage)	15 (75%) 0 3 (15%) 1 (5%) 1 (5%)	5 (50%) 1 (10%) 2 (20%) 1 (10%) 1 (10%)	21 (70%) 1 (3%) 5 (17%) 2 (7%) 1 (3%)	-
**Mean Post-Op** **MSTS Score** (min–max)	26.7 (15–30)	27.6 (25–30)	27.0 (15–30)	-
**Follow-Up** (min–max)	60.4 months (21–89)	53.5 months (20–72)	58.1 months (20–89)	-

## Data Availability

The data that support the findings of this study are available from the corresponding author upon reasonable request.
